# Sensing Cognitive Responses Through a Non-Invasive Brain–Computer Interface

**DOI:** 10.3390/s26061892

**Published:** 2026-03-17

**Authors:** Hristo Hristov, Zlatogor Minchev, Mitko Shoshev, Irina Kancheva, Veneta Koleva, Teodor Vakarelsky, Kalin Dimitrov, Dimiter Prodanov

**Affiliations:** 1 Institute of Information and Communication Technologies, Bulgarian Academy of Sciences, 1113 Sofia, Bulgaria; zlatogor.minchev@gmail.com (Z.M.); kyn4@abv.bg (I.K.); teodorvakarelsky@gmail.com (T.V.); dimiter.prodanov@iict.bas.bg (D.P.); 2 Plovdiv University “Paisii Hilendarski”, 4000 Plovdiv, Bulgaria; mitko.shoshev@gmail.com; 3 High School of Mathematics and Natural Sciences “Acad. Nikola Obreshkov”, 8000 Burgas, Bulgaria; veneta_kolevaa@abv.bg; 4 Faculty of Telecommunications, Technical University of Sofia, 1756 Sofia, Bulgaria; kld@tu-sofia.bg

**Keywords:** mental workload, multimodal physiological sensing, non-invasive brain–computer interface (BCI), repeated-measures ANOVA, EEG alpha/theta ratio

## Abstract

Cognitive stress, also known as mental workload, constitutes a central topic within the field of psychophysiology due to its role in modulating attention, autonomic regulation, and stress reactivity. Furthermore, it bears direct relevance to practical monitoring systems that employ non-invasive sensing techniques. This study investigates whether a multimodal, non-invasive measurement setup can detect systematic physiological differences between Resting periods and short episodes of cognitive load within the same individuals. Additionally, it explores the capacity of such a system to differentiate tasks characterized by varying cognitive demands. A sequential, within-subject protocol was employed, comprising five consecutive phases (rest 1, Stroop, rest 12, subtraction, rest 3), during which five modalities were recorded concurrently: EEG, heart rate (HR), galvanic skin response (GSR), facial surface temperature, and oxygen saturation (SpO_2_). Beyond phase-wise inspection of time-series data, an exploratory assessment of similarity across participants was conducted using correlation coefficients. The maximum cross-participant correlations observed were 0.88 (HR), 0.90 (GSR), 0.83 (facial temperature), and 0.77 (SpO_2_); however, these correlations were used only as exploratory descriptors of inter-individual similarity and did not imply a significant phase effect. For inferential analysis, phase-wise epoch means were evaluated through one-factor repeated-measures ANOVA. The heart rate exhibited a robust main effect of phase (F(4, 32) = 10.5862, p_GG = 0.01044, ηp^2^ = 0.5696), with higher HR observed during cognitive load epochs (e.g., 77.841 ± 11.777 bpm at rest 1 versus 83.926 ± 14.532 bpm during subtraction). The relatively large standard deviation reflects variability between subjects rather than variability within epochs. Regarding processed baseline-referenced GSR, the omnibus phase effect was not statistically significant under the conservative Greenhouse–Geisser correction; therefore, GSR was interpreted as exploratory in this dataset. Facial temperature and SpO_2_ likewise did not show statistically significant omnibus phase effects under Greenhouse–Geisser correction (e.g., SpO_2_: p_GG = 0.1209). EEG-derived measures provide supplementary central evidence of task engagement; entropy variations within an approximate dynamic range of 0.2 to 0.8 were observed, and the α/θ ratios demonstrated nearly a twofold distinction between rest and cognitive load epochs across different leads.

## 1. Introduction

The mental state, stress levels, work capacity, as well as the timing and methods of rest, are increasingly recognized as significant individual factors. Stress and compromised mental health may lead to physical illnesses, depression, loss of concentration and productivity, and strained social relationships. Consequently, proper rest and effective time management are crucial for maintaining quality of life and overall well-being. In recent years, systems designed to assess current psychological states have gained broader application: they support the diagnosis and monitoring of mental disorders (e.g., depression and anxiety), enable organizations to evaluate employee stress and psychological stability, and assist in identifying students’ needs to ensure that educators can offer appropriate support. Mental stress assessment has become increasingly relevant across industry, healthcare, education, security, and sports, where understanding how the brain responds to varying types and durations of stress is a shared priority. To investigate mental activity, multimodal systems are frequently employed to measure diverse parameters during cognitive task performance [1]. Key physiological markers—including EEG, pulse and heart activity, oxygen saturation in the blood, and related autonomic measures—are utilized, as their variations can aid in evaluating stress factors and predicting their influence on the current mental state. The assessment of stress offers opportunities to prevent undesirable workplace situations and to enhance work and learning processes [2].

Brain–computer communication systems have been developed for diverse scientific and medical applications, including restoring quality of life for individuals with motor disorders or neuromuscular injuries and enhancing performance in professional activities. Brain–computer interfaces (BCIs) based on electroencephalography (EEG) offer a non-invasive means of capturing brain activity through scalp electrodes. EEG is considered safe and provides high temporal resolution, making it suitable for real-time applications; it is extensively employed in BCI systems due to its cost-effectiveness, portability, and sensitivity to rapid changes in neuronal activity [3]. To enhance performance and reliability, multimodal BCIs increasingly integrate EEG with additional physiological signals. In complex and dynamic environments, research has utilized combinations such as functional near-infrared spectroscopy (fNIRS), EEG, electrocardiography (ECG), and galvanic skin response (GSR) to examine stress and cognitive load [4]. The integration of EEG with GSR (also known as electrodermal activity, EDA) constitutes a well-established approach for capturing both neural and autonomic activity; GSR reflects sympathetic modulation of skin conductance and correlates with arousal or stress responses that are largely outside conscious control. Extending EEG + GSR with infrared thermography (facial surface temperature) further broadens multimodal monitoring by incorporating a non-contact measure associated with autonomic regulation and peripheral perfusion. Such parallel sensing offers complementary physiological evidence that may enhance the assessment of cognitive and emotional states and increase robustness amidst real-world noise and individual variability.

While the motivation for multimodal stress and workload monitoring is strong, the recent literature shows that the field is methodologically diverse and often difficult to compare across studies. A “fusion-centric” systematic review of EEG-based stress research (covering studies up to early 2025) emphasizes the wide variability in pipelines, feature sets, fusion strategies, and validation practices, and repeatedly notes challenges in standardization and reproducibility [5].

A complementary systematic review concerning mental workload in human–robot collaboration (HRC) similarly reports considerable heterogeneity even within a single application domain: EEG, cardiac measures, and EDA are frequently employed; ocular measures (such as fixations, saccades, pupil size, and blink metrics) have gained increasing prominence; and temperature measures continue to be relatively underutilized [6]. These syntheses imply that advancement is contingent not solely upon the development of “better classifiers,” but also on more precise construct definitions (clarifying what is being measured), improved ground-truth methodologies, and more uniform reporting practices concerning synchronization, preprocessing, and quality assurance.

Evidence from applied domains demonstrates both the potential and the limitations of multimodality. In remote assistance for highly automated vehicles, time-resolved indicators such as eye metrics (e.g., pupil dilation), tonic electrodermal activity, and cardiac measures are responsive to workload variations through auditory n-back tasks; however, the accuracy of person-independent multi-class classification remains modest at approximately 58% for three classes, thereby emphasizing a persistent gap in generalization between within-person detectability and cross-person application [7]. A related driving study employing auditory n-back during simulated driving integrates EEG spectral power, EOG/eye measures, ECG-derived HRV, EDA features, driving performance metrics, and NASA-TLX, reporting systematic subjective workload increases alongside modality-specific sensitivities to task difficulty [8]. It is important to note that multimodality is not inherently advantageous: a comparative study utilizing a driving simulator has demonstrated that eye-gaze/pupillometry can surpass other modalities. Furthermore, the fusion of modalities may not enhance, and can even diminish, the performance of the most effective unimodal baseline. This suggests that supplementary sensors may introduce artifacts and misaligned temporal structures without providing additional valuable information [9]. Conversely, a study on web-browsing workloads demonstrates that using a comprehensive multimodal set—comprising EDA, ECG, PPG, EEG, temperature, and pupil dilation—can achieve high-accuracy differentiation of workload levels, provided that protocol constraints and labeling are consistent with ocular responses under controlled lighting conditions [10]. Collectively, these results suggest that the justification for fusion should be based on empirical evidence through ablation and complementarity analysis, rather than being assumed to be beneficial by default.

Despite the proliferation of available datasets, fundamental issues of labeling inconsistency and limited comparability remain largely unresolved, constraining the progress they are meant to enable. The ADABase dataset is a comprehensive multimodal dataset of cognitive load in autonomous driving paradigms. It integrates physiological and behavioral data such as ECG, EDA, EMG, PPG, respiration, skin temperature, eye tracking, and facial video/action units. Additionally, it includes subjective workload assessments (NASA-TLX), performance results, and a biochemical marker (salivary cortisol) [11]. Its broad scope enables systematic examination of multimodal fusion strategies, missing data patterns, and distribution shifts across tasks. However, it also highlights a common challenge: workload “targets” are frequently composite constructs that combine subjective appraisal, performance, and physiological reactivity [11]. The MMD-MSD dataset, collected in an office-like environment, combines synchronized vision with wearables (Empatica E4: BVP, EDA, skin temperature, accelerometry) and structured tasks like Stroop and posture changes, facilitating analysis that connects cognition, behavior, and ergonomics [12]. These resources are valuable not only for benchmarking models but also for clarifying practices related to synchronization, annotation, and quality control, which directly impact reproducibility and generalization.

Recent modeling efforts focus more on short time frames and near-real-time inference. One method converts peripheral biosignals, such as ECG and EDA, into image-like formats, uses copula-based Granger causality graphs to model dependencies between signals, and employs a lightweight capsule network classifier. This approach demonstrates excellent results even with very brief windows, approximately 1 s, on standard datasets [13]. While these results are promising, they also highlight a translational gap: methods validated on curated benchmark datasets do not necessarily apply to smaller, noisier, more diverse experiments unless sensor quality, missing data, and individual differences are explicitly addressed in both modeling and evaluation.

Within industrial HRC, neuroergonomics studies reveal both potential and complexity in integrating central and peripheral measures. A smart-factory protocol alters task difficulty (such as a Stroop-like primary task with a secondary task), robot speed, and payload, recording EEG band-power features and fNIRS hemodynamics. It also includes NASA-TLX and behavioral performance measures to determine if physiological indicators can estimate or replace traditional subjective and performance outcomes [14]. This approach aligns with the HRC review’s focus on enhancing standardization and combining various evidence types to boost interpretability and practical deployment.

An additional interpretive issue is that physiological changes attributed to “workload” might be influenced by affective appraisal. A large driving simulator study connects high-risk scenarios to anger, fear, and anxiety and breaks down workload into visual, auditory, cognitive, and psychomotor components, showing that emotional intensity can vary together with workload indicators and thus complicate establishing ground truth and comparing results across studies [15]. This is important whenever stress, arousal, and cognitive demand occur together rather than separately in experiments.

Building on this broader psychophysiological motivation, foundational BCI research has shown that non-invasive EEG can enable α/θ communication and control, serving as essential methodological components and validation methods for modern mental-state monitoring [16]. Community benchmarks like the BCI Competition III tested alternative approaches under similar conditions, highlighting the importance of standardized evaluation for meaningful progress [17]. Early successes with EEG-based control in non-invasive BCIs demonstrated the feasibility of translating brain signals into continuous control variables [18], while later work on machine learning for real-time, single-trial EEG analysis connected traditional BCI goals with mental-state monitoring needs [19]. Asynchronous paradigms—such as EEG-based virtual keyboards—underscored the importance of accurate state detection without fixed trial limits [20]. Other asynchronous BCI uses, like brain-actuated wheelchairs and virtual environment wheelchair control, focused on robustness and continuous operation in realistic settings [21,22]. Ultimately, BCI control of robotic and prosthetic devices integrated these advances into a wider range of applications, supporting multimodal systems that combine EEG with autonomic measures for practical, real-world monitoring [23].

Against this background, the present study is designed as a controlled, within-subject study to determine whether brief episodes of cognitive stress (mental workload) cause measurable physiological changes within the same individuals and whether tasks with different cognitive demands can be distinguished. A sequential within-subject protocol is used, consisting of five phases (rest 1, Stroop, rest 2, subtraction, rest 3), while five modalities are recorded simultaneously: EEG, heart rate (HR), galvanic skin response (GSR), facial surface temperature, and oxygen saturation (SpO_2_). In addition to phase-by-phase examination of time-series data, inter-individual similarity is analyzed using correlation coefficients, showing that correlations across participants can be high for some modalities but may also be negative, reflecting significant individual differences in baseline levels and response patterns. For inferential purposes, phase-based epoch means are assessed using one-factor repeated-measures ANOVA within an omnibus hypothesis framework: H0 assumes equal modality means across phases, while H1 suggests that at least one phase mean differs, with significance indicating a phase effect without specifying which phases differ unless tested with planned contrasts.

This positioning directly addresses several recurring gaps identified in recent reviews, datasets, and applied research: (i) it highlights within-subject detectability within a unified, sequential timing framework; (ii) it considers inter-individual variability as an explicit focus rather than leftover noise; and (iii) it includes under-characterized but practical channels—most notably facial thermography (facial surface temperature) and SpO_2_—which have not yet shown consistent incremental value beyond HR and GSR in the literature.

The study examines whether brief episodes of cognitive stress—defined as mental workload—cause measurable, systematic changes in physiological responses within the same individuals, consistent with standard psychophysiological methods for studying attention, emotion regulation, and stress reactivity. A multimodal, non-invasive sensing setup is used, allowing simultaneous recordings of EEG, heart rate (HR), galvanic skin response (GSR), facial thermography (facial surface temperature), and oxygen saturation (SpO_2_). The main goal is to see if these measures can distinguish resting periods from cognitive load periods, and if the overall multimodal response profile is sensitive to differences between the two task types (Stroop versus mental subtraction). For statistical testing, an omnibus repeated-measures design is applied separately to each modality across five sequential phases (rest 1, Stroop, rest 2, subtraction, rest 3): the null hypothesis (H0) states that phase means are equal, while the alternative hypothesis (H1) suggests at least one phase mean differs. As a comprehensive (omnibus) test, statistical significance indicates a phase effect but does not specify which phase pairs differ unless planned contrasts are used.

While extensive research has focused on multimodal workload and stress monitoring, studies that simultaneously combine EEG, HR, EDA/GSR, facial infrared thermography, and SpO_2_ within a single repeated-measures protocol—and that align different signals on consistent analysis intervals—remain relatively scarce. Therefore, this study acts as a pilot within-subject investigation, employing a unified timing scheme (rest 1–Stroop–rest 2–subtraction–rest 3) to analyze phase effects statistically and assess individual differences, rather than aiming to develop a universally applicable classifier.

## 2. Subjects, Materials and Methods

### 2.1. Subjects

The study was conducted under the oversight and approval of the Ethical Committee of TU, Sofia. Healthy volunteers were recruited by placing adverts at the premises of the host institutions. Informed consent was obtained from all subjects involved in the study. Ten healthy volunteers participated in the study (7 male and 3 female, average age 43.8 years, all right-handed) after signing an informed consent form.

### 2.2. Study Protocol

**Figure 1:**
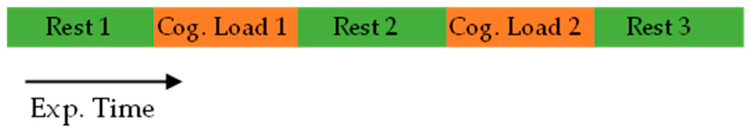
Sequence of the experimental protocol.

The experiment includes five consecutive phases: an initial three-minute baseline rest (rest 1), a three-minute Stroop test, a two-minute break (rest 2), a subtraction task designed to increase cognitive load, and a final three-minute recovery rest (rest 3). The *rests* states were with eyes closed, whilst the *cog. loads* were with eyes opened. Measurements are taken every 30 s, starting at the beginning of the first rest and ending after the last rest. Signals are collected continuously at the device’s native sampling rates. However, the multimodal analysis is based on predefined, synchronized 30 s analysis windows. Therefore, “every 30 s” indicates the analysis resolution—when features for each modality are extracted and aligned—not the raw sampling frequency of the signal’s sensors. This structure alternates periods of cognitive load with recovery intervals, allowing for direct comparison of physiological responses during different states and capturing short-term recovery dynamics. The setup is designed to ensure high signal quality, minimize noise and motion artifacts, and support reproducibility, while keeping discomfort minimal for participants. This makes it suitable for research on non-invasive brain–computer interfaces and multimodal mental-state monitoring. Figure 1 visualizes the stages of the protocol.

### 2.3. Stroop Test

The Stroop task is a neuropsychological test of attention and cognitive control that assesses the ability to inhibit cognitive interference that occurs when the processing of one feature of a stimulus influences the simultaneous processing of another attribute of the same stimulus [24,25]. The task requires naming the color in which the word is written, not the word itself denoting a color. This is a relatively complex task that requires the simultaneous involvement and work of both hemispheres of the brain, and in the case of this test, the so-called “hemisphere conflict” can also be examined. Physiologically, the left hemisphere is dominant in most people, which is why they relatively easily name the word denoting a color, but experience difficulty when they have to name the color in which the corresponding word is written. This leads to the use of greater mental effort, a redistribution of tone from the left to the right hemisphere, and in some cases a person may show an increased level of stress. When mental effort increases, the temperature emitted by the prefrontal areas of the cerebral cortex usually rises as well. The requirement for increased mental activity can also be demonstrated through visualization using the appropriate IR sensor or thermal camera with precise beam targeting of the prefrontal areas.

### 2.4. Arithmetical Test

The second test is related to mental arithmetic, with the task being to subtract numbers mentally. In this way, activation is achieved predominantly in the left hemisphere (the left prefrontal area of the cerebral cortex) in subjects within the normal range. Performing this task may lead to a slight increase in certain values of cardiac activity or galvanic skin conductance, which can be associated with the individual psychological characteristics of the subjects being studied. Mental arithmetic is also used in the educational sphere [26].

### 2.5. Data Processing

To test for a relationship between the application of the tests and the participants’ responses, we calculated the full combination of 180 correlation coefficients between the results of all pairs of participants for each monitored parameter, for the entire study period, with accuracy to the second decimal place. The exception is EEG, where we applied a different approach. The levels of these correlation coefficients will allow us to establish the informativeness of the individual monitored parameters.

The anonymized data records have been further processed in MATLAB R2011b environment. Initially the data was separated into five epochs spread totally in 14 min, discriminating three *rests* (rest 1 and rest 3, both of 3 min each and rest 2—with 2 min duration) and two cognitive loads—*cog. loads* (cog. load 1 and cog. load 2, both of 3 min. each). Further, data was processed using bandpass Butterworth 12 dB/Oct digital filter with 0.5–70 Hz bandwidth and zero-phase shift. A followed processing with Chebyshev notch at 50 Hz ± 5 Hz with 18 dB/Oct was also added for supply network hums eliminating. According to [27,28], bandpass filtering has been organized for the theta (4–7 Hz) and alpha (8–13 Hz) rhythms [29] with the same bandpass Butterworth 12 dB/Oct digital filter.

Though the experimental setup and electrodes mounting was reliable enough, a few sparse artifacts mostly related to eyes blinking, saccade movements or other unplanned muscle contractions have to be handled via the already mentioned bandpass and notch filtering. Additionally, in order to achieve a better accuracy regarding the signals non-stationarities, wavelet denoising has also been implemented by Daubechies 4 core [30], focusing mostly on the studied subjects’ awaken EEG activity frequency band. With this two-phase EEG processing, good, artifact-free signals have been provided for further analysis.

The obtained results were used for averaged Alpha entropy H’ calculation, taking the Shannon approximation [31]. Other measures, like approximated entropy [27] and Kolmogorov’s entropy [32], have also been tested but showed a higher demand for computational and time resources in comparison with the selected one for the presented experiment duration. Results were taken for both epoch types (*rests* vs. *cog. loads*) and EEG leads.

### 2.6. EEG

Electroencephalography (EEG) was used to record cortical electrical activity via electrodes mounted on the scalp surface. The technique captures micro-voltage fluctuations arising from synchronous postsynaptic potentials in cortical pyramidal neurons, providing millisecond-level temporal resolution of neural oscillations underlying perception, cognition, and motor control [33]. EEG signals were acquired using BrainBit Flex8, with 8 electrodes placed according to the international 10–20 system. Its combination of high temporal resolution, portability, and non-invasiveness makes EEG well suited for real-time cognitive workload assessment in the experimental paradigms employed here [34,35,36].

### 2.7. Galvanic Skin Response (GSR)

The galvanic skin response (GSR) signal is primarily used for the measuring of emotional arousal and physiological reactions, often applied in psychology, biofeedback, usability testing, and stress research. In the present study, GSR was recorded, using Mind-Reflection device of VERIM© & VERIM LAB Light 1.2 software environment, using an elastic band with two electrodes from the front phalanges of the middle and ring fingers of the non-dominated subjects’ hands, while staying in a calm, resting position. As some subjects have drier fingers, Ten20 conductive paste was used additionally in order to achieve lower montage resistance. The results were stored in ASCII format file and further processed (see Section 3) [37,38].

### 2.8. Infrared Thermography

Infrared thermography is a remote method for detecting and tracking thermal radiation. It can be used to measure and study changes in surface temperature from a distance. Thermography is based on radiometry in the infrared spectrum. Thermal energy is electromagnetic in nature, with the distribution of energy described by Planck’s law of radiation from an ideal black body. The following equation shows a mathematical expression of this law, according to which anybody that has a temperature other than absolute zero emits radiation constantly, with the spectrum depending on its surface temperature [39].

(1) $$L\left(\lambda ,T\right)=\frac{2hc^{2}}{\lambda^{5}}\frac{1}{exp\left(\frac{hc}{\lambda kT}\right)-1},\;[W\;m^{-2}\;Hz^{-1}\;sr^{-1}]$$

where *T* represents the absolute temperature, *h* is Planck’s constant (6.626 × 10^−34^ J⋅s), *c* denotes the speed of light (3.0 × 10^8^ m/s), and *k* indicates Boltzmann’s constant (1.381 × 10^−23^ J/K), ԑ denotes emissivity, $\sigma =5.67037.10^{-8}\;Wm^{-2}K^{-4}$ denotes Stefan–Boltzmann constant.

Real bodies have different structures, different surface temperatures, and therefore emit differently. In order to be able to compare them and make different calculations related to their energy, the emissivity coefficient has been introduced. It is an individual parameter and characterizes the emissivity of each real body. The following equation shows a mathematical formula for its calculation.

(2) $$\varepsilon \left(\lambda ,T\right)=\frac{M_{\lambda }\left(\lambda ,T\right)}{M_{\lambda ,Blackbody}\left(\lambda ,T\right)},$$

where $M_{\lambda }\left(\lambda ,T\right)$ is the spectral radiative power output [W·$m^{-2}$·μ$m^{-1}$] and $M_{\lambda ,Blackbody}\left(\lambda ,T\right)$ is the ideal black body spectral radiative power output [W·$m^{-2}$·μ$m^{-1}$].

The normal body temperature of humans is about 37 °C, making the operating range between 8 µm and 14 µm suitable for measuring such surface temperature.

Infrared thermography was applied to measure facial skin temperature, with consistent region-of-interest (ROI) selection across all participants being critical to the validity of the results. The ROI was defined as a small area of the forehead above the inner edge of the eyebrow and below the midline of the forehead. This region was selected for two reasons: it overlies a richly vascularised area sensitive to autonomic thermoregulatory changes, and it remains free of hair, which acts as a thermal insulator and would otherwise attenuate the measured surface temperature signal.

Since participants were not fully stationary during recording, minor head movements—even small ones at the camera-to-participant distance used—were sufficient to displace the observed area onto hair, eyebrows, EEG electrodes, or entirely off the participant’s surface, each of which would introduce artifactual temperature values. To ensure that temperature data were consistently extracted from the same anatomical location throughout the recording, ROI tracking was performed manually for each participant.

### 2.9. Pulse Oximetry

Peripheral oxygen saturation (SpO_2_) and heart rate were recorded as cardiovascular indicators of cognitive load using a Beurer PO 30 pulse oximeter. Pulse oximetry is a non-invasive technique that provides continuous, real-time measurement of both parameters with high accuracy and minimal participant burden, making it well suited for monitoring physiological responses during task performance. Elevated mental load has been associated with increased cerebral oxygen consumption [40,41,42], and heart rate has been shown to vary systematically during mental arithmetic and other cognitively demanding tasks [43].

### 2.10. Setup

**Figure 2:**
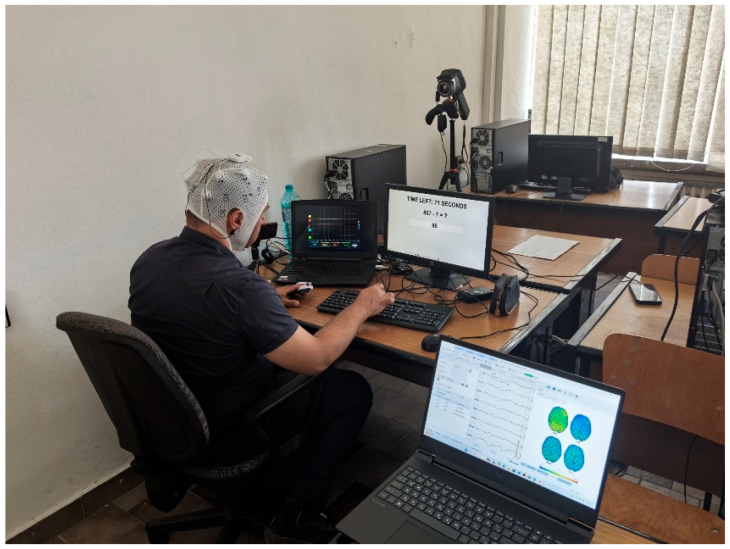
A moment from the study and the experimental setup.

The experimental setup is a multimodal EEG-based non-invasive BCI system with integrated parallel measured indicators: heart rate (HR), galvanic skin response (GSR), facial temperature and oxygen saturation (SpO_2_). The experiments were performed in a normal office, quiet environment, while subjects were comfortably sitting in front of a computer screen, interfering with the experimental setup via desktop computer with a keyboard and a mouse interface in the feedforward direction and visual feedback via the setup monitor test results’ responses (see Figure 1). Ten healthy volunteers have been studied (7 male and 3 female, average age 43.8 years ± 10.69, all right-handed) after signing an informed consent form. The thermal camera is orthogonally aligned to the facial plane, positioned about one meter from the participant, at a height slightly above eye level, with its angular displacement not exceeding ±15° in order to avoid errors related to emissivity. To avoid inaccuracy in the thermal camera data, the region of interest (ROI) of the thermal image is localized in the same field for all participants, and in order to average the sensor noise, it includes at least 5 × 5 pixels. In order to avoid introducing artifacts and noise into the thermal and electrodermal signals, the light and temperature in the room are controlled and constant. The pulse oximeter is placed on the tip of the index finger of the left hand. The GSR sensor is placed on the ring finger and little finger of the participant’s left hand. The dominant right hand remains free for task-related activities. All sensors are connected to a central laptop that manages data collection and synchronization in real time using a shared system clock. Thermographic images are captured with a FLIR E40 thermal camera and processed using FLIR Tools [44]. The brain activity has been recorded from the subjects’ scalp with a BrainBit Flex8 EEG wearable device within F3, F4, C3, C4, P3, P4, O1, and O2 leads (according to international 10–20 Jasper system, with sampling frequency—fs = 250 Hz), using a spring-loaded gold-plated dry electrodes set and a supportive elastic cap with reference electrodes as ear clips and ground handled internally [45,46]. Before each recording, the electrodes impedance within the studied subject scalp was measured, assuring good contact for all leads and high amplitude of the recorded signals. All measuring and recording activities have been organized in BrainBit NeuroREC 3.0 environment installed on HP Victus Gaming Laptop 16-s0000nu platform (having the following key parameters: 32 GB RAM, AMD Ryzen™ 7 processor, 1TB SSD, NVIDIA^®^ GeForce RTX™ 4060 video card and Windows 11 operation system) and the results have been saved in a raw form in EDF format for further processing. Figure 2 shows a participant photographed from behind and the experimental setup of the study.

All 10 volunteers took part in the experiment. However, one participant had several missing values in the heart rate (HR) series, even though the other sensor channels for that participant were available and usable. Since the phase-wise inferential analysis required complete data across the variables and phases analyzed, the rmANOVA was conducted on the 9 participants with full data. Similarly, correlation coefficient analyses used the same 9 complete-case participants to ensure consistency and prevent mixing different effective sample sizes. After excluding the data from one participant, we rechecked the participants’ mean values and the associated variability (43.7 ± 11.26).

Participant-level time-series figures mainly serve to illustrate temporal behavior and compare response patterns qualitatively. Therefore, they do not necessarily include all participants in each figure. Conversely, the statistical results in the manuscript are based on the processed datasets used for each analysis, reflecting the explicitly defined effective sample size for each case test.

The raw measurements were stored in a CSV format with semicolon-separated fields and decimal commas for temperature, so a specific parsing and cleaning process was used. For repeated-measures analysis, the raw data were summarized into epoch means, resulting in one value per subject × phase for each modality.

## 3. Results

EEG analytical results, concerning averaged entropy H’ statistics are presented in Table 1 and averaged alpha/theta ratios statistics in Table 2. Additional generalized findings are organized into three subsections for clarity: (i) phase-wise descriptive statistics (mean ± SD) summarized in Table 3, (ii) inferential effects within subjects using repeated-measures ANOVA with effect sizes and Greenhouse–Geisser correction, as shown in Table 4, and (iii) exploratory comparison of cross-participant similarity through correlation ranges to highlight inter-individual differences. In participant-level plots, each curve represents one participant. If a participant’s data is missing in a figure, the caption specifies the number of included participants and provides the reason (e.g., sensor dropout or unusable segment after artifact screening) to clarify data availability and selection.

### 3.1. Facial Surface Temperature

Throughout the study period, the similarity in facial surface temperature between participants showed significant variation: the lowest pairwise correlation coefficient was −0.63, the average correlation was around −0.01 (indicating almost no overall agreement), and the highest correlation reached 0.83. This reflected clear differences between individuals in baseline levels and how they responded.

**Figure 3:**
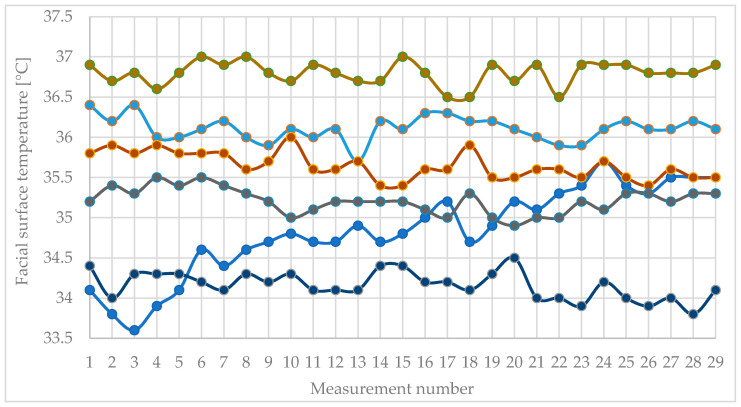
This figure displays the variation in surface skin temperature, with each curve representing data from an individual participant. For clarity, only selected participants are shown to illustrate the overall time-course patterns.

Figure 3 shows the values of the facial surface temperature of a large proportion of the participants for the entire period of the study.

The surface temperature of the face follows a behavior that is partially repeated as a type in different participants. It is clearly seen that at the end of the first break, the temperature decreased in most measurements. Peaks are also seen during mental workload during both tests (between the 7th and 13th measurements and between the 17th and 23rd). There are exceptions, of course, but there are noticeable trends for facial temperature to increase during mental exertion and decrease during rest. It can be seen that the speed of these temperature variations is different for different participants.

### 3.2. Heart Rate

Throughout the entire study, similarity in heart rate (HR) between participants varied significantly: the lowest pairwise correlation coefficient was −0.58, the average is 0.38, and the highest reached 0.88. This indicates notable differences among individuals in baseline levels and response patterns, even though some participant pairs show moderately consistent trends.

**Figure 4:**
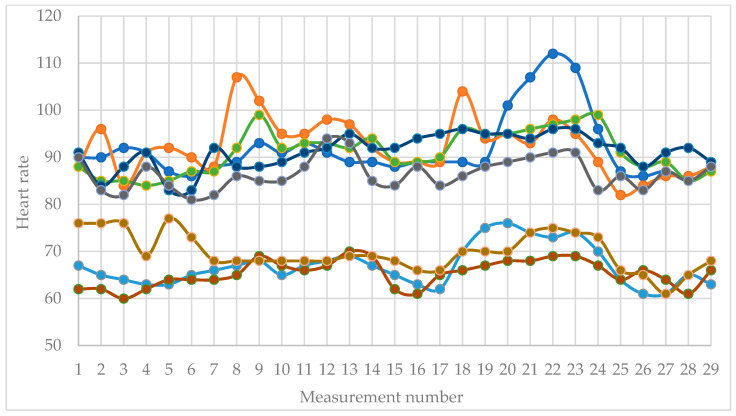
Heart rate values. Each curve represents the measurements recorded from one participant. The figure includes only selected participants for illustrative visualization of the time-course patterns.

Figure 4 shows the heart rate values of almost all participants for the entire study period.

This measurement also clearly shows the increase in heart rate during the tests that require mental activity and the correspondingly lower values during the breaks. Again, there are differences, which are mainly visualized as different peak heights in the different participants. Measurement number 7 is the last of the first resting period. During the first test, peaks in the heart rate of some of the participants are clearly visible (measurements with numbers 8, 9, 12 and 13). During the second rest, drops in the heart rate levels of most participants are clearly visible (measurements with numbers 14, 15 and 16). Subsequent measurements with numbers from 18 to 23, which are during the second test and in the zone of the second mental load, again clearly show the increase in the heart rate levels and are visible on the diagram as peaks of different heights. At the end of the experiment during the last rest (measurements with numbers from 24 to 29), the diagram clearly shows the decrease in the heart rate values, which corresponds to the rest state. At the end of the experiment, the heart rate values fell to the relative values of heart rate at rest.

### 3.3. Oxygen Saturation

Across the full study period, cross-participant similarity in oxygen saturation (SpO_2_) showed wide dispersion: the minimum pairwise correlation coefficient reaches −0.74, the average correlation was approximately 0.02 (i.e., near zero overall agreement), and the maximum correlation reached 0.77, indicating substantial inter-individual differences in baseline levels and response dynamics.

**Figure 5:**
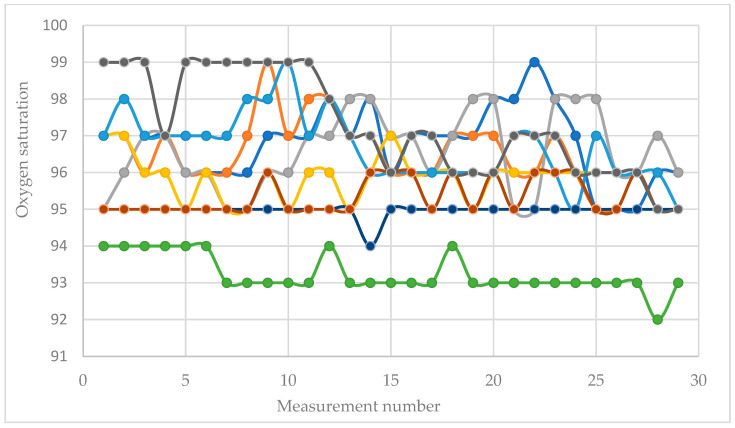
Oxygen saturation values over the entire study period. Each curve represents the measurements recorded from one participant. The figure includes only selected participants for illustrative visualization of the time-course patterns.

Figure 5 shows the oxygen saturation values of most participants for the entire study period.

Oxygen saturation measurements show relatively stable values. Some of the diagrams show small drops during periods of mental stress, but the stable behavior of this value indicates its low informativeness in this type of research.

### 3.4. Galvanic Skin Response

Throughout the study, cross-participant similarity in galvanic skin response (GSR) showed significant variability, with pairwise correlation coefficients ranging from −0.58 to 0.90 and an average of 0.26. These values indicated substantial differences between individuals in baseline conductance and response patterns, and they were mainly reported as descriptive context rather than as evidence of a strong group-level effect.

**Figure 6:**
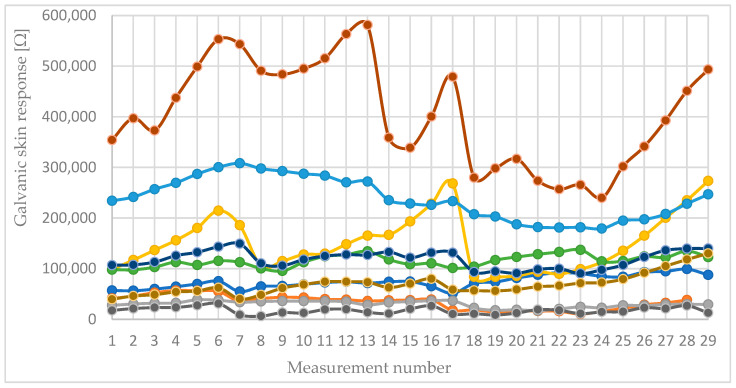
Values of the galvanic skin response of all participants for the entire study period. Each curve represents the measurements recorded from one participant.

Figure 6 visualizes the values of the galvanic skin response of all study participants.

### 3.5. Electroencephalography (EEG)

**Figure 7:**
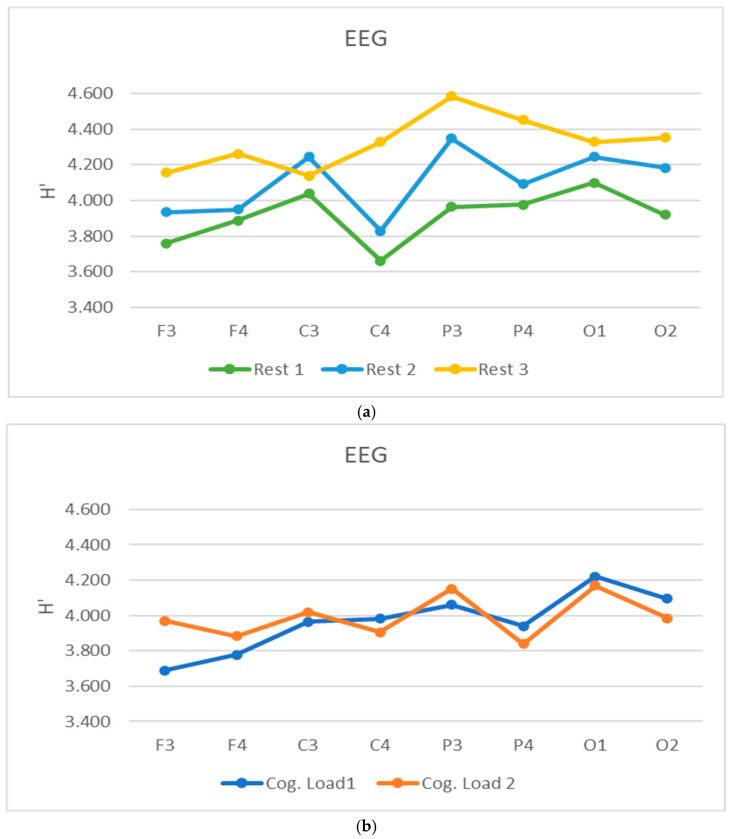
Averaged entropy H’ for all studied subjects, spread amongst all eight EEG leads for *rests*—rest 1, rest 2, rest 3 (**a**) and *cog. loads*—cog. load 1 and cog. load 2, (**b**) epochs of the experiment.

**Figure 8:**
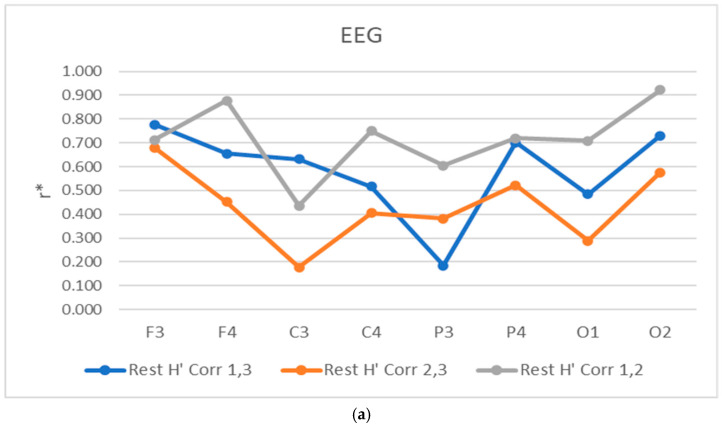

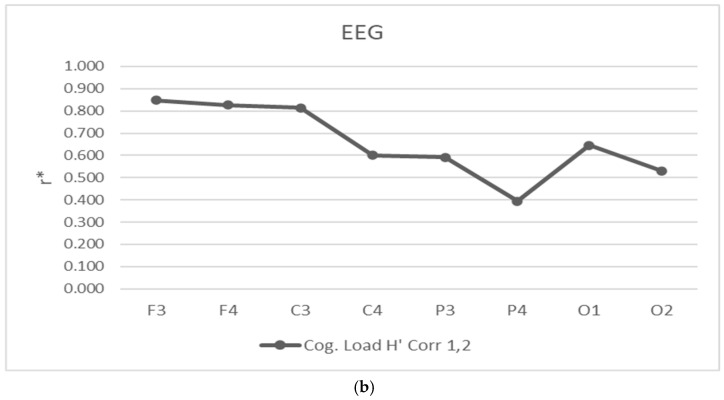
Averaged correlation coefficients r* for all studied subjects, spread amongst the eight EEG leads for rests: (**a**)—rest H’ Corr 1, 3, rest H’ Corr 2, 3, rest H’ Corr 1, 2 and cog. loads, (**b**)—cog. load H’ Corr 1, 2 epochs of the experiment.

Additional correlation coefficients r* have been calculated [27] for both epochs, regarding the E’ entropy. The aggregated findings concerning these analyses are depicted in Figure 7 and Figure 8, as follows:

As it is clear from the averaged entropy H’ results for both *rests* and *cog. loads*, the rests episodes are somewhat oscillating with all brain areas, giving higher values after each cognitive load episode. These dynamic changes are clearly observed for both parietal and occipital brain areas, whilst giving slight domination to the left hemisphere leads (P3, O1) vs. right ones (P4, O2). The lowest results are observed with central–right leads—C4, and both frontal and central left ones are showing higher entropy values.

The situation concerning both *cog. loads’* episodes (load 1 and load 2) are giving almost equal dynamics for all recording leads, whilst giving some dominance to the left hemisphere (C3, P3 and O1 leads) in comparison to the right ones for the load 2 episode. It should also be noted here that the measured differences are not so high (in the interval of 0.2–0.8 from the overall H’ entropy dynamics).

As for the correlations’ r* dynamics, the aggregated results demonstrate a frontal dominant correlation, concerning the fronto-central zones during cognitive loads identification. In comparison, the parietal correlations, though still significant, are giving lower results for the right hemisphere. Similar results are also observed in the occipital zones. Regarding the *rests*’ parts of the experiment, the results are more dynamic, giving aggregated and synchronized responses to the frontal but left hemisphere (for F3 leads), whilst the right ones of the frontal and central zones demonstrate lower correlations (for leads F4 and C4), also adding the same response for C3 leads. In addition to these findings, the lowest correlations are also observed with the left parietal leads of P3 and partially occipital ones with O1. The rest of the responses from P4 and O2 are giving much better correlation performance. The most significant results are observed between the first two *rests*, and less between the second and third ones.

To complement the primary analysis and enable meaningful pairwise comparisons despite the limited sample size, additional statistical tests were applied to the averaged entropy results (H’). This includes the Mann–Whitney U test, Wilcoxon signed-rank test, paired permutation test, and two summary indices—mean and Combined Tendency Score (CTS)—as reported in Table 1. These tests were included for completeness; however, the non-stationary nature of EEG signals limits the interpretability of entropy-based statistics when considered in isolation.

The findings presented above do not demonstrate a sufficiently clear discrimination between rest and cognitive load episodes, which is consistent with the well-established complexity of EEG signals [29] and their sensitivity to emotional context [31]. Nevertheless, the statistical tests reveal a moderate initial effect for the first cognitive load episode (load 1), interpretable as the subjects’ initial experimental activation, characterized statistically as a “moderate upward tendency.” This tendency persists following rest 2 and becomes most prominent in the third episode pair (load 2 and rest 3), with both episodes sustaining the upward trend.

**Figure 9:**
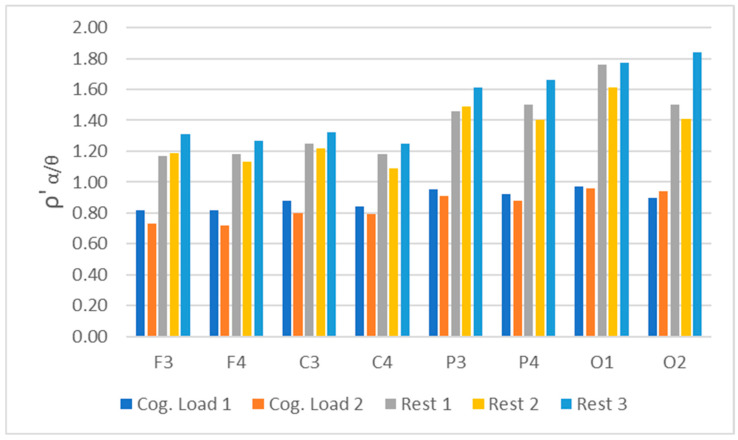
Averaged ρ’—alpha/theta ratio for all studied subjects, spread amongst the eight EEG leads for *rests*—rest 1, rest 2, rest 3 and *cog. loads—cog. load* 1 and cog. load 2 epochs of the experiment.

As these results show some oscillation and are constrained by reliance on the alpha frequency band alone, the analysis was extended to incorporate the theta band. Following the approach of [36], averaged alpha/theta ratios (ρ’ α/θ) were computed for all rest and cognitive load episodes and are presented in Figure 9.

The obtained results have shown a clear discrimination capability within all EEG leads and experiment *rests* and *cog. loads* episodes, providing almost doubled averaged ratios, ρ’, between them, whilst keeping strong statistical significance and upward tendency.

### 3.6. Cross-Modal Interpretation of Phase Effects

To maintain a balanced within-subject design for testing, only participants with complete data across all five phases were included, resulting in nine balanced subjects. Because raw GSR values can vary greatly between individuals in baseline and dynamic ranges, the analysis also employed a processed GSR measure to improve comparability across subjects, based on subject-wise min–max normalization and baseline referencing to rest 1 (which becomes roughly zero by design). An optional smoothing step was added but was effectively turned off in the reported run (window length = 1).

The statistical analysis was executed using a standalone Python 3.12.2 script. For each modality (HR, GSR_rel, facial temperature, and SpO_2_), the script fits a one-factor RM-ANOVA model with phase as the within-subject factor (five levels: rest 1, Stroop, rest 2, subtraction, rest 3) and subject as the repeated-measures identifier. The output includes the F-statistic with its degrees of freedom, the uncorrected *p*-value, partial eta-squared as an effect size, Greenhouse–Geisser epsilon (εGG), and a GG-corrected *p*-value as a conservative approach for inference. The calculations are performed using stats models with AnovaRM.

In the RM-ANOVA, the inferential question is posed as a comprehensive test to determine whether the experimental phase has any consistent effect on a specific physiological variable. For each modality (HR, processed GSR, facial temperature, and SpO_2_), the same pair of hypotheses is tested:

Null hypothesis (H0): The mean value of the modality remains the same across all five phases (rest 1, Stroop, rest 2, subtraction, rest 3). Essentially, H0 suggests that any differences observed between phases are due to random variation rather than the experimental procedure.

Alternative hypothesis (H1): At least one phase has a mean value that differs from the others. Importantly, H1 does not specify which phase differs nor the direction of the difference.

As an omnibus test, significance indicates a phase effect without specifying which phase pairs differ. It is important to note that the reported standard deviations represent between-subject variability of the phase-wise epoch means (i.e., differences in baseline levels and individual reactivity), rather than measurement noise within epochs (Table 3).

Regarding heart rate (HR), the average level increased during the cognitive load epochs compared to rest. The mean HR elevated from 77.841 ± 11.777 bpm at rest 1 to 80.630 ± 14.538 bpm during the Stroop task, returned close to baseline during rest 2 (77.889 ± 14.136 bpm), and reached its peak during the subtraction task (83.926 ± 14.532 bpm). The subsequent resting period (rest 3) reverted to values near the initial resting state (77.167 ± 13.037 bpm). Although mean differences between phases are modest relative to the relatively large standard deviations (indicating substantial baseline variability among participants), the overall pattern is consistent with increased cardiac activation during subtraction in this sample.

Regarding the processed galvanic skin response measure (GSR_rel), the phase-wise averages remained highly variable and did not exhibit a stable monotonic trend across phases. Rest 1 was approximately zero (~0 ± ~0), as the GSR metric is subject-normalized and baseline-referenced to rest 1. Relative to this baseline, the mean values were 0.0144 ± 0.1990 for Stroop, −0.0324 ± 0.2310 for rest 2, −0.2328 ± 0.4416 for subtraction, and 0.0442 ± 0.3982 for rest 3. The standard deviations were large relative to the means, indicating substantial heterogeneity in electrodermal responses across individuals and/or variability in the direction and magnitude of reactivity under cognitive load.

Facial temperature exhibited only modest fluctuations between phases, with mean values remaining confined within a narrow range. The average temperature was 35.270 ± 1.140 °C at rest 2 1, 35.296 ± 0.902 °C during Stroop, 35.447 ± 0.848 °C at rest 3 2, 35.322 ± 0.817 °C during subtraction, and 35.465 ± 0.902 °C at rest 3. Although rest 2 and rest 3 display slightly higher mean temperatures compared to rest 1, these differences are minimal relative to the between-subject standard deviations, consistent with slower thermal dynamics and prominent individual differences over the brief epochs examined.

Oxygen saturation (SpO_2_) demonstrates minimal fluctuations across phases. The mean SpO_2_ values were 96.095 ± 1.409% at rest 1 1, 96.259 ± 1.696% during Stroop, 95.861 ± 1.282% at rest 2, 95.981 ± 1.324% during subtraction, and 95.333 ± 1.074% at rest 3. Variability was comparatively low; however, mean differences across phases were also very small (less than one percentage point), supporting the interpretation of SpO_2_ as predominantly stable during short cognitive load tasks in healthy individuals.

The RM-ANOVA results quantify whether these apparent differences are statistically significant (Table 4).

For heart rate, the phase effect was strong: F(4, 32) = 10.5862, *p* = 1.4037 × 10^−5^ (uncorrected), with a large effect size (ηp^2^ = 0.5696). The Greenhouse–Geisser correction yields εGG = 0.2616 and a corrected *p* = 0.01044, confirming the significance of the phase effect. This indicates that HR systematically varies across the five phases.

For the processed GSR_rel, the overall phase effect was not significant: F(4, 32) = 2.1798, *p* = 0.09368 (uncorrected), ηp^2^ = 0.2141, εGG = 0.3437, and pGG = 0.1661. Despite a moderate effect size, the sample size and variability do not support rejecting the null hypothesis that phase means are equal for this measure.

Facial temperature shows no evidence of a phase effect: F(4, 32) = 0.6532, *p* = 0.6289 (uncorrected), ηp^2^ = 0.07549, εGG = 0.3010, and pGG = 0.4665. These results suggest that temperature either changes slowly or is heavily influenced by individual differences during short epochs, rather than displaying sharp, phase-locked shifts.

For SpO_2_, the uncorrected test is marginal (F(4, 32) = 2.7331, *p* = 0.04605, ηp^2^ = 0.2546), but the Greenhouse–Geisser correction reduces the significance (εGG = 0.3382, pGG = 0.1209). Under a conservative approach, this indicates no robust phase-related modulation of SpO_2_, and any observed differences should be interpreted with caution.

Using a conservative inference rule based on GG correction, the overall conclusion is that a statistically significant phase effect is only present for heart rate, while no significant omnibus phase effects are observed for GSR_rel, facial temperature, or SpO_2_.

## 4. Discussion

Considering the pilot nature of this study with a small within-subject sample, the interpretations are intentionally conservative and based solely on empirically supported data from the current dataset. Effects related to phases are analyzed using a repeated-measures approach, while modalities beyond the primary autonomic measures are considered secondary or exploratory, due to notable individual differences and modality-specific responses during short epochs. Therefore, strong assertions about “reliability,” “validation,” or specific neurophysiological mechanisms are avoided unless backed by formal statistical evidence. The discussion instead highlights observed trends, variability, and how consistently effects appear under cautious inference.

The analysis of the collected physiological data (facial temperature, heart rate, oxygen saturation, and galvanic skin response) across ten participants reveals distinct patterns and inter-individual variability in response to cognitive load. The following observations, derived from the dataset, complement the EEG findings and provide a broader understanding of the physiological correlates of mental workload.

The findings presented here may be tentatively interpreted as reflecting a sustained neural response to cognitive engagement. Cognitive load episodes appear associated with increased EEG theta band activity, while rest states are characterized predominantly by increased alpha band activity—a pattern consistent with established accounts of task-related cortical dynamics. The observed dynamics of averaged entropy H’ can be interpreted within the same framework, as shifts toward higher frequencies during load episodes are known to correspond to increased signal entropy.

It should be noted that the analysis was intentionally restricted to the alpha and theta bands as simple, reliable, and statistically interpretable metrics for discriminating eyes closed rest states from eyes open cognitive load states. The remaining EEG frequency bands—beta and gamma—were not examined here but represent a natural avenue for deeper investigation of the underlying neural mechanisms driving the observed dynamic changes.

1. GSR time series displayed varied, participant-specific fluctuations during rest and task periods. Changes in amplitude varied significantly among individuals in both direction and size. Correlation analysis also showed wide variability, with pairwise coefficients ranging from −0.58 to 0.90 and an average of 0.26, indicating limited overall agreement and high dependence on each person’s baseline conductance and response patterns. Importantly, conservative rmANOVA analysis found no statistically significant overall phase effect for GSR. Therefore, GSR was considered an exploratory channel in this dataset and not viewed as a reliable group-level measure of cognitive load.
2. Heart rate (HR) shows a reliable increase during cognitive tasks.
  A clear and consistent trend of increased heart rate was observed during both cognitive tasks (Stroop and arithmetic) compared to the rest periods. The data shows an average increase of approximately 5–15 BPM from resting baseline to the peak of cognitive effort.
  The correlation coefficients for HR between participants were positive on average (0.38), with a maximum of 0.88. This suggests a more uniform directional response (increase during load) across the cohort compared to other measures like temperature or SpO_2_.
  Observation: HR offers the clearest group-level evidence of phase-related modulation in this dataset, consistent with the expected cardiovascular response to cognitive stress.
3. Facial surface temperature demonstrates complex and biphasic patterns.
  Contrary to a simple uniform increase, facial temperature dynamics were more complex. A common pattern observed in multiple participants was an initial decrease in temperature at the onset of a cognitive task, followed by a subsequent rise as the task continued.
  The correlation between participants was the lowest on average (−0.01), with a wide range from −0.63 to 0.83. This indicates that while some participants showed strong correlated patterns (e.g., a clear temperature rise with load), others showed inverse or uncorrelated responses, making it a less reliable standalone metric without individual baseline.
  Facial thermography detects dynamic physiological changes, but its response varies greatly among individuals in this protocol and should be interpreted cautiously when analyzing group data. The biphasic pattern (initial drop followed by an increase) may reflect rapid vasoconstriction followed by increased cerebral blood flow and metabolic heat.
  Oxygen saturation (SpO_2_) shows minor fluctuations with low inter-participant consistency. The SpO_2_ levels remained within the normal physiological range (95–99%) in all participants throughout the experiment. While subtle decreases (1–2%) were sometimes recorded during cognitive tasks, these changes were not consistent across all subjects or all task periods.
  This is supported by the correlation data, which shows an average correlation near zero (0.02) between participants, indicating no consistent, synchronized pattern of SpO_2_ change in response to the cognitive loads in this study.
  Peripheral oxygen saturation appeared largely stable during short cognitive load epochs in this context and did not demonstrate significant phase-related modulation at the group level.
4. High inter-participant variability underscores the need for personalized assessment.
  The calculated correlation matrices for all non-EEG parameters consistently show a wide spread of correlation coefficients (from strongly negative to strongly positive) for every possible pair of participants.
  This finding strongly suggests that while the direction of change for parameters like HR is often consistent (e.g., HR generally goes up), the specific pattern, magnitude, and timing of each individual’s physiological response is unique.
  The significant inter-participant variability across measured channels highlights challenges for “one-size-fits-all” interpretation and supports the use of within-subject designs and conservative inference in small-sample multimodal studies.
5. The RM-ANOVA results quantify phase-related modulation in the peripheral physiological measures within a rigorous within-subject framework. This methodology is highly appropriate for the current protocol, as it explicitly accounts for repeated measurements from the same individuals and separates systematic phase effects from substantial inter-individual differences in baseline levels and overall variability. In this dataset, the standard deviations of the epoch means indicate that between-subject dispersion is often substantial relative to phase-to-phase shifts in the means; thus, RM-ANOVA offers a principled method for testing whether the observed changes are consistent within participants across phases, rather than being influenced primarily by baseline differences among participants. A significant omnibus F-test supports the conclusion that at least one phase differs significantly in mean value, while a non-significant result suggests that any apparent differences are relatively small compared to within-subject consistency and residual variability at the group level. EEG-derived findings are regarded solely as supplementary contextual evidence for task engagement and are not incorporated into the formal inferential claims presented in this section.

### 4.1. Summary of Principal Findings

Among the autonomic measures, heart rate (HR) demonstrated the clearest sensitivity to task demands; it increased during both cognitive loads and was more pronounced during the more difficult arithmetic task. Galvanic skin response (GSR) showed variable, subject-specific task-related changes but did not exhibit a statistically significant omnibus phase effect under conservative correction and was therefore interpreted as exploratory in this dataset. Facial temperature did not exhibit strong phase-locked shifts across the group and instead followed slower, individual-specific patterns during the protocol. SpO_2_ remained relatively stable, as expected during seated cognitive tasks, thus serving as a physiological baseline. EEG was summarized by alpha/theta ratios across eight leads per epoch; this central measure is theoretically expected to decrease with increased workload, providing a complementary perspective to the peripheral measures.

### 4.2. Fast vs. Slow Channels

The data correspond with established time scales. Fast changes include HR (beat-to-beat and short windows), phasic GSR (event-related peaks), and EEG alpha/theta, which can fluctuate within seconds of task start. Intermediate changes involve tonic GSR, showing gradual drifts over minutes. Slow variations are seen in facial temperature, which accumulates over minutes rather than reacting immediately to task events. Understanding these time scales is essential for selecting appropriate windowing methods—using event-locked windows for HR, GSR-phasic, and EEG, and epoch averages and trends for temperature.

### 4.3. On Integrating Heterogeneous Modalities

Multimodal integration is valuable but should be used sparingly. No single channel suffices for all participants and tasks; combining central (EEG) with peripheral (HR, GSR, temperature) signals improves robustness and provides convergent evidence (in this dataset) (e.g., HR increase and GSR-phasic increase alongside EEG α/θ decrease during load). However, integration should happen in stages:

1. Normalize each subject individually (e.g., z-score or min–max); for GSR, separate phasic from tonic components and normalize to rest 1.
2. Extract compact epoch-level features per modality (e.g., HR mean and HRV; GSR peak rate/amplitude and tonic level; facial temperature mean and change from previous rest; EEG α/θ per region).
3. Perform late fusion (weighted z-sum or a small multivariate model) after validating each modality independently.
4. Conduct cross-modal checks (repeated-measures correlations, cross-correlation for lead–lag).

Beyond the omnibus phase tests, modality-specific patterns are described as exploratory observations without proposing extra post hoc hypotheses.

### 4.4. Role of EEG Alpha/Theta

The averaged alpha/theta ratios were calculated across eight leads per epoch to assess cortical states. Typically, α/θ ratios decrease with increased workload. In this dataset, the alpha/theta metric serves two complementary purposes: (i) confirmatory, by testing whether α/θ (rest) is greater than α/θ (Stroop) and α/θ (rest 2) exceeds α/θ (subtraction), preferably using a within-subject mixed model; and (ii) convergent, by examining correlations between α/θ and HR and GSR across epochs, expecting negative correlations with HR and phasic GSR during task load. Even if power is limited at α/θ bands, directional trends enhance the interpretation of results across multiple modalities.

### 4.5. Correlation Structure and Lead–Lag

Simple epoch-wise correlations are useful but should be supplemented with repeated-measures correlation to prevent inflation caused by between-subject differences and lagged analyses, such as cross-correlation, to investigate if EEG changes occur before autonomic responses. With n = 10, such timing assessments should stay descriptive but can inform future study designs, like using shorter windows around task onsets.

### 4.6. Limitations

The sample size is small (ten participants), which limits the ability to detect small effects and restricts the complexity of multivariate models. Epoch lengths are inconsistent, and the slow dynamics of temperature can obscure task contrasts. Large between-subject variability in GSR requires normalization. These limitations led to the use of mixed models, conservative feature sets, per-subject scaling, and planned contrasts.

### 4.7. Implications and Future Work

The results propose a practical approach for non-invasive workload assessment: use HR as a quick, reliable marker; include normalized phasic GSR to detect higher loads; utilize EEG α/θ for central validation; consider facial temperature as a slow-changing context indicator; and keep SpO_2_ as a measure of stability. Future research should involve larger samples, standardize event-locked windows, and pre-register concise feature sets. With increased data, it becomes possible to analyze a latent workload factor that combines EEG and autonomic features using Bayesian or SEM methods, and to evaluate how well the model generalizes through cross-validated predictions.

In summary, multimodal sensing is justified even at small scales when each modality is processed on its natural time scale, normalized per subject, and interpreted within a confirmatory mixed-model framework. HR and normalized GSR indicate load (and task difficulty), EEG α/θ provides central convergence, facial temperature reflects slow drift, and SpO_2_ confirms physiological stability, together supporting the viability of a non-invasive, multimodal approach to current psychological state assessment.

## 5. Conclusions

The correlations discussed in this study mainly serve as a descriptive complement to the within-subject phase analyses. Correlation coefficients, whether between different modalities or between participants for the same modality, do not inherently demonstrate discriminative ability or causal relationships. Nonetheless, they are valuable for understanding two key aspects of multimodal, non-invasive BCI monitoring. Firstly, they reveal how strongly various physiological channels tend to co-vary under a shared protocol, which helps determine whether modalities offer overlapping or complementary information. Secondly, correlations across participants offer a succinct way to illustrate the variability in temporal response patterns among individuals, highlighting a major challenge in creating generalizable mental-state monitoring systems and emphasizing the need for cautious interpretation and personalized baselining.

Combined with the phase-wise within-subject results, these descriptive correlation patterns suggest the broader potential of non-invasive multimodal BCI systems to detect relevant psychophysiological changes during brief cognitive load periods. Although the current pilot data do not allow for definitive population-wide conclusions, they indicate that multimodal sensing may help assess mental state when used carefully and within suitable analytical methods. Gaining a better understanding of how physiological responses vary together and differ across individuals could improve monitoring of everyday stress and workload, which are known to impact well-being, focus, and productivity. Consequently, these findings offer a structured empirical foundation for future, larger-scale studies on psychological stress, using more standardized data collection and predefined outcomes.

The study not only focused on peripheral sensing but also included an EEG processing pipeline designed to offer additional central nervous system insights into task engagement. EEG data underwent bandpass and mains-notch filtering, with alpha (8–13 Hz) and theta (4–7 Hz) bands extracted to generate compact, window-level descriptors across the eight recorded leads. To mitigate the effects of sparse, non-stationary artifacts common in this pilot setup, wavelet denoising was applied. The cleaned signals were then summarized using entropy-based descriptors and alpha/theta ratios, serving as practical metrics to compare rest (eyes closed) and cognitive load (eyes open) epochs. These EEG summaries were used cautiously as supportive context rather than definitive evidence of discriminative performance.

For the primary within-subject analysis, phase-wise epoch means were tested using a one-factor repeated-measures ANOVA with a conservative Greenhouse–Geisser correction. Based on complete-case data (n = 9), heart rate was the only modality showing a statistically significant omnibus phase effect across the five phases. In contrast, baseline-referenced GSR, facial temperature, and SpO_2_ did not reach significance after correction. Therefore, the main conclusion from this pilot data was that HR demonstrated strong phase-related changes under cognitive load, while the other peripheral measures should be considered exploratory or context-dependent, as their apparent variations were small compared to individual differences.

In order to build successful practices for studying the influence of cognitive stress, as well as methodologies for overcoming stressful conditions, taking into account the difficulty of such studies and the many influencing factors, we would like to point out the need for additional research.

## Figures and Tables

**Table 1:** Averaged entropy H’ statistical tests results for the studied subjects, spread amongst all eight EEG leads for *rests* (rest 1, rest 2, rest 3) and *cog. loads* (load 1, i.e., Stroop test and load 2—Subtraction experiment) epochs of the experiment.

Comparison	Mann–Whitney *p*	Wilcoxon *p*	Permutation *p*	Mean *p*	CTS	Interpretation
load 1 → rest 1	0.4619	0.3828	0.4991	0.447	0.553	Moderate upward tendency
load 1 → rest 2	0.2268	0.0547	0.1432	0.141	0.859	Strong upward tendency
load 2 → rest 3	0.0011	0.0078	0.0012	0.0037	0.997	Extremely strong upward tendency

**Table 2:** Averaged ρ’—alpha/theta ratio statistical tests results for the studied subjects, spread amongst all eight EEG leads for *rests* (rest 1, rest 2, rest 3) and *cog. loads* (load 1, i.e., Stroop test and load 2—Subtraction experiment) epochs of the experiment.

Comparison	Mann–Whitney *p*	Wilcoxon *p*	Permutation *p*	Mean *p*	CTS	Interpretation
load 1 → rest 1	0.0009	0.0078	0.0068	0.00518	0.99482	Extremely strong upward tendency
load 1 → rest 2	0.0009	0.0078	0.0077	0.00548	0.99452	Extremely strong upward tendency
load 2 → rest 3	0.0002	0.0078	0.0086	0.00552	0.99448	Extremely strong upward tendency

**Table 3:** Phase-wise descriptive statistics (mean ± SD).

Modality	Rest 1	Stroop	Rest 2	Subtraction	Rest 3
Heart rate (bpm)	77.841 ± 11.777	80.630 ± 14.538	77.889 ± 14.136	83.926 ± 14.532	77.167 ± 13.037
GSR_rel (a.u.)	~0 ± ~0	0.0144 ± 0.1990	−0.0324 ± 0.2310	−0.2328 ± 0.4416	0.0442 ± 0.3982
Facial temperature (°C)	35.270 ± 1.140	35.296 ± 0.902	35.447 ± 0.848	35.322 ± 0.817	35.465 ± 0.902
SpO_2_ (%)	96.095 ± 1.409	96.259 ± 1.696	95.861 ± 1.282	95.981 ± 1.324	95.333 ± 1.074

**Table 4:** One-factor repeated-measures ANOVA results (phase effect).

Modality	F(4, 32)	p (Uncorrected)	ηp^2^	εGG	pGG (Greenhouse–Geisser)	Conclusion (GG)
Heart rate (HR)	10.5862	1.4037 × 10^−5^	0.5696	0.2616	0.01044	Significant
GSR_rel	2.1798	0.09368	0.2141	0.3437	0.1661	Not significant
Facial temperature	0.6532	0.6289	0.07549	0.3010	0.4665	Not significant
SpO_2_	2.7331	0.04605	0.2546	0.3382	0.1209	Not significant

## Data Availability

The data sets presented in this study are available on request from the corresponding author.

## Abbreviations

The following abbreviations are used in this manuscript:

ANOVA	Analysis of Variance
RM-ANOVA	Repeated-Measures Analysis of Variance
BCI	Brain–Computer Interface
bpm	Beats per Minute
BVP	Blood Volume Pulse
CSV	Comma-Separated Values
ECG	Electrocardiography
EDA	Electrodermal Activity
EDF	European Data Format
EEG	Electroencephalography
EMG	Electromyography
EOG	Electrooculography
fNIRS	Functional Near-Infrared Spectroscopy
FLIR	Forward-Looking Infrared
fs	Sampling Frequency
GG	Greenhouse–Geisser (Correction)
εGG	Greenhouse–Geisser Epsilon
pGG	Greenhouse–Geisser-Corrected *p*-Value
ηp^2^	Partial Eta-Squared
GSR	Galvanic Skin Response
GSR_rel	Baseline-Referenced, Subject-Normalized GSR Measure (Processed)
HRC	Human–Robot Collaboration
HR	Heart Rate
HRV	Heart Rate Variability
IR	Infrared
NASA-TLX	NASA Task Load Index
PPG	Photoplethysmography
ROI	Region of Interest
SEM	Structural Equation Modeling
SpO_2_	Peripheral Oxygen Saturation
a.u.	Arbitrary Units
dB/Oct	Decibels per Octave
H0 / H1	Null / Alternative Hypothesis
H′	Shannon Entropy
ρ′ (α/θ)	Alpha/Theta Ratio (EEG Index)
r*	Correlation Coefficient
TU-Sofia	Technical University of Sofia
